# Investigation of mechanical properties and performance of automotive brake pads

**DOI:** 10.1038/s41598-025-15116-3

**Published:** 2025-09-01

**Authors:** Mahmoud A. Essam, Noha M. Abdeltawab, Ahmed Y. Shash, Mostafa M. El-Sayed

**Affiliations:** 1 Mechanical Engineering Department, Higher Technological Institute (HTI), 10th of Ramadan City, Egypt; 2 Faculty of Engineering and Materials Science, German University in Cairo, Cairo, Egypt; 3 Mechanical Design and Production Engineering Dept, Faculty of Engineering, Cairo University, Giza, 12316 Egypt; 4 Manufacturing and Production Engineering Dept, Modern Academy for Engineering and Technology, Cairo, Egypt

**Keywords:** Braking pad, Automotive, Wear, Coefficient of friction (COF), Engineering, Mechanical engineering

## Abstract

This study evaluates the performance of three powder metallurgy-based brake pad formulations (BP1, BP2, and BP3) by examining mass loss, hardness, braking force, coefficient of friction (COF), noise, and vibration under 5 and 8 bar pressures. BP1 exhibited the highest braking force (640.99 N) and COF (0.3873) at 8 bars, with improvements of 7.7–13.6% and 4.4–6.8% over BP2 and BP3, respectively. However, this came with the highest mass loss (1.2 g/h), noise (17.5 dB), and vibration (0.743 m/s²), attributed to three-body abrasive wear from SiC and ZrO₂ particles.BP3 demonstrated the lowest mass loss (1.07 g/h, ~ 20% lower than BP1), noise (14.8 dB), and vibration (0.571 m/s²), making it suitable for quiet, long-life applications. BP2 showed balanced behavior across all parameters. Hardness values were 46 HRC (BP1), 38 HRC (BP2), and 44 HRC (BP3), aligning with observed braking forces and structural compactness.

## Introduction

Materials used for brake friction are essential to the braking system. During the braking process, they use friction to transform the kinetic energy of a moving vehicle into thermal energy^1^. To retain a vehicle’s braking characteristics, the optimum brake friction material should have a constant coefficient of friction under a variety of operating situations, including applied loads, temperature, speeds, braking mode, and dry or wet conditions. In addition, it should have a number of desirable qualities, including low mass loss, high thermal stability, low noise, and resistance to heat, water, and oil^2^. It should also not harm the brake disc. However, having all of these desired qualities is very impossible. Therefore, in order to meet some standards, some other requirements must be compromised. Generally, every friction material composition has distinct wear-resistance properties and frictional behaviors^1,3^.

Friction material is made up of several components, each of which serves a specific purpose, such as enhancing friction characteristics at both low and high temperatures, boosting strength and rigidity, extending life, decreasing porosity, and lowering noise^4^. The physical, mechanical, and chemical characteristics of the brake friction materials to be developed may alter if the types of elements or their weight percentages in the formulation change^4^.

Brake pads come in two different structural varieties: asbestos and non-asbestos^5^. The resistance to temperatures at which the brake pads can still function varies between the two of them. While non-asbestos brake pads are more heat resistant to braking temperatures of 350 °C because cellulose and other fibers can reduce heat better than asbestos fibers, asbestos brake pads will not occur or will not work at a braking temperature of 200 °C, which leads to an accident rate that will occur quickly^6^.

The ingredients used to make brake pads must always be readily available and not go extinct. The mango seed, often known as a paddle, is one of them. In Indonesia, one million tons of mango seed waste are produced annually, but at least two hundred thousand tons could be utilized. Crude protein, oil, ash, crude fiber, and carbs are all found in mango seeds^7^. Given the aforementioned issues, brake pads must be manufactured using a blend of brass and magnesium oxide and mango seeds, a natural fiber material^8,9^.

### Literature review

In cars, brake pads are disc parts made of friction materials bonded to the surface of steel plates. In order to continually grip and hold wheels in order to slow down or halt their motion, they are attached to the surface facing the brake disc and placed in the wheel assembly^2^. Its purpose is to control the speed of a moving vehicle by converting kinetic energy to thermal energy through friction and releasing the generated heat into the environment. The majority of car brake pads on the market are categorized as non-asbestos organic (NAO), metallic, or semi-metallic compounds^10,11^. Binders, fillers, structural materials, and frictional additives are examples of friction materials. Semi-metallic friction materials are ones that incorporate metal powders, whereas asbestos friction materials are those made of asbestos. Asbestos-free non-asbestos friction materials are those that don’t contain asbestos^5^. For drum brakes, brake shoes are housed inside a drum such that they are pushed outward and up against the drum when the brakes are applied. Disc brakes and drum brakes work similarly, with the exception that disc brakes are exposed to the elements, whilst drum brakes are enclosed^12^.

The lateral force, often known as the friction force, between two rubbing surfaces is one of the most significant and fascinating scientific phenomena associated with brake systems. If a block is pulled across a horizontal floor, the friction force between the two surfaces equals the lateral force needed to move the block^11^.

Typically, organic pads are made up of a variety of components. Occasionally, as many as twenty or twenty-five components are employed^13^. Among these elements is a binder, which creates a thermally stable matrix and holds the other elements together. Rubber is frequently added to thermosetting phenolic resins to enhance their damping capabilities^13^.

Materials that are structural and give mechanical strength. Metal, carbon, glass, and/or kevlar fibers are typically utilized, with various mineral and ceramic fibers being employed less frequently. Asbestos was the most widely used structural fiber prior to its prohibition in the middle of the 1980s^9,12^.

Fillers, mostly for cost reduction but also for improved manufacturing efficiency. Various minerals, including vermiculite and mica, are frequently used. Another popular filler is barium sulfate^14^.

A cerametallic friction pad (CMDE) was created via the powder metallurgy process and confirmed through a conventional SAE J 661 standard laboratory test using a Chase Testing machine, subsequently followed by a field test. A comparison was made with an imported OE (CMOE) pad. In the chase test, CMDE underperformed in fade-1 by 25% relative to the reference value, although met the criteria for fade-II and recovery characteristics. From a wear perspective, the exact wear rates of CMDE and CMOE are relatively similar. In the field test, CMDE exhibited 11.1% greater wear than CMOE, although demonstrated 20% reduced wear in the mating component. The decrease in surface roughness of CMDE following the field test demonstrated its engagement properties through the deformation of asperities. Adhesive wear scoring marks were evident along the sliding trajectory of the pressure plate and the sintered friction pad^15,16^.

Frictional additives are used to manage the mass losss of the pad and disc and to guarantee steady frictional qualities. The coefficient of friction is stabilized, mainly at high temperatures, using solid lubricants like graphite and other metal sulphides. Both the coefficient of friction and disc wear are increased by abrasive particles, usually silica and alumina. By eliminating iron oxides and other undesirable surface coatings from the disc, the latter aims to provide a more defined rubbing surface^17^.

Molybdenum disulfide serves as an effective solid lubricant due to the fracture of its basal plane while sliding, which preserves its lubricating properties, hence minimizing wear and ensuring frictional stability^18,19^.

Friction stability is an essential variable in how well friction materials perform. When tested under different operating conditions, such as speed, pressure, temperature rise, and area of real contact, it is the friction material’s capacity to maintain a constant or steady µ^20^. The role of abrasives as a friction stabilizer was proposed by some studies, while others asserted the role of solid lubricants^21^. Commercially available abrasives include mild abrasives such green chrome oxide, barite, magnetite, magnesium oxide, cryolite, and others, as well as severe abrasives like alumina, silicon carbide, quartz, and zirconium silicate^1^.

One of the most important features is the abrasives’ hardness (Mohs scale 7–9), which is higher than that of the cast-iron disc (Mohs scale 5–6). Numerous academics have examined how abrasives affect the brake pad’s or linings’ wear and friction stability. Brake pads were made using four different abrasives: SiC, quartz, MgO, and zircon. The tribological analysis revealed that one of the key factors in raising the friction level, wear resistance, and stick-slip phenomena was fracture toughness^22^. Additionally, research on the use of nanometer-sized abrasives in brake pads revealed that they significantly improved wear resistance and friction compared to conventional abrasives^5^0.16 Boz and Kurt^23^ examined how much Al_2_O_3_ was used in the formulation of the friction material and found that adding more Al_2_O_3_ improved the frictional stability and wear resistance.

Brake pads using graphite as lubricants and Al_2_O_3_ and boron carbide as abrasives were created by Öztürk et al.^24^. They discovered that graphite combined with boron carbide increased fade resistance and friction stability. Kim et al.^25^ investigated brake pads containing various abrasive particles, including silicon carbide, zircon, quartz, and magnesia. They proposed that the abrasive’s fracture toughness was a key factor in vibration-related problems during braking.

Jang and Kim^25^ investigated the interaction between the abrasive zircon and the solid lubricant antimony. They found that zircon induced torque variation during braking applications and eliminated the paralyzed coating on the mating surface^1^. On the interface between the pad and the disc, abrasive particles operate in two-body or three-body abrasion modes in friction materials.

Shape, volume percentage, and the strength of the abrasive-resin bonding are some of the elements that considerably influence these modes and the transition between these two abrasion modes during the dry sliding, which greatly affects the performance^26^. An analysis of the wear resistance and friction efficiency of brake pads containing abrasive particles revealed that fracture toughness is one of the key characteristics that affects how well abrasives work in brake pads^27^.

**Fig. 1:**
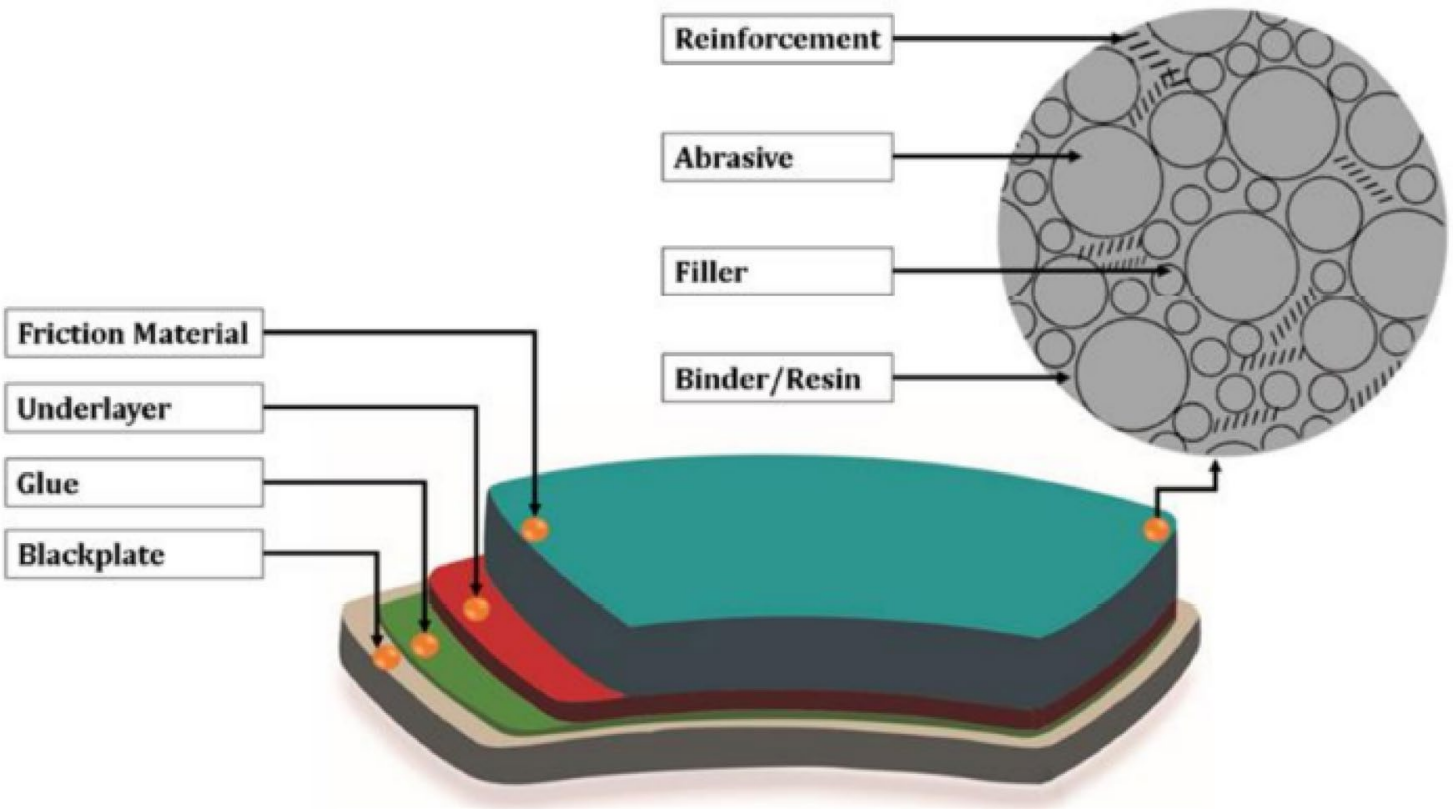
Explain the layers of braking pads^28^.

Multiple layers make up brake pads as shown in Fig. 1^28^. The underlayer, which sits between the friction material and the backplate, provides the adhesive that binds the friction material to the other layers. The main purpose of the underlayer is to lessen vibrations brought on by friction materials coming into contact with the disk. The backplate allows the brake pads to continue moving on the caliper guides by providing the necessary stiffness. Some industries use particular interference shims to reduce the amount of unneeded noise when braking. The crucial layer on the brake pads is the friction substance that comes into direct contact with the disc when braking. Each of the elements used to make this substance was created with a specific purpose in mind^28^.

Binders, reinforcement, fillers, and abrasives make up the friction material of brake pads, Fig. 1. The polymers that hold the various parts of the pads together are called binders. This material needs to be lightweight, resistant to high temperatures and abrupt temperature changes, and have a stable and high coefficient of friction. A fibrous substance called reinforcement is added to the binder to improve its mechanical properties^29^. The kinds of reinforcing materials utilized have a big impact on how long the brake pads last. One of the best reinforcing fibers is asbestos. However, a new material is needed because of its hazardous nature. While abrasive substances are used to alter the coefficient of friction, fillers are used to fill in the spaces between the brake pads’ other components. For example, as a result of their hardness, steel, refractory oxides, cast iron, quartz, or silicates are used as additives to increase the friction coefficient between the disc and the brake pads. Increasing the friction coefficient extends the life of the brake pads^30^.

Several criteria can be used to categorize the materials used to make brake pads. The substance’s function in the braking process is the most crucial. There are binders, fillers, additives, and abrasives based on this criterion^31^.

The tube that contains all of the pad’s components is called the binder. This material needs to have a low mass (the binder typically makes up 20% of the pad volume), a high and consistent coefficient of friction, and resilience to high and quickly changing temperatures^36^. Additionally, the material must not react with any other pad component, as this could alter the material’s overall properties or cause the composite to delamination, which would significantly reduce the braking system’s efficacy. Typically, silicone resin or epoxy are used to make the binder^32,33^.

One or more fibrous materials serve as reinforcement, enhancing the binder’s mechanical qualities and boosting its strength. Since the longevity and resistance of the brake pad are greatly influenced by the types of reinforcement materials used, the choice cannot be made at random. Asbestos was a great reinforcement fiber in the past. But because of its detrimental qualities^34^, a substitute had to be found, which is not an issue anymore because a variety of materials may be utilized effectively for this purpose^35,36^.

Fillers are utilized to fill up the gaps that exist between the brake pad’s other components. Since they might account for as much as 10% of the brake pad volume, it is crucial to use the appropriate material. Vermiculite, perlite, mica, barium sulfate, and calcium carbonate are the most often used fillers because of their low cost, durability to high temperatures, and inability to react with other brake pad ingredients^37^.

The coefficient of friction can be modified, either raised or decreased, through the use of abrasives. Additives such as steel, cast iron, silicates, flame-resistant oxides, and quartz are utilized to enhance the coefficient of friction between the brake pad and disc, hence prolonging the pad’s operational lifespan due to their hardness. Moreover, the effect is amplified by the stickiness of the disc material, especially with metals^38^. Additionally, the materials produce contact zones, which are the primary locations of friction between the two parts^37^. Unfortunately, high temperatures are produced as a result of friction in the contact zones.

For this reason, lubricants are applied, which often increase the pad’s thermal conductivity. In addition to keeping the friction parts from overheating, lubricants enhance the removal of energy from the contact region^39^. Graphite and metallic sulphates (such copper or tin) are the most widely used lubricants. The pad’s content (about 10% of volume produces the optimum effects) and lubricant particle size determine how lubricating they are^40^.

The authors’ objectives are to evaluate and compare the performance of three pow-der-metallurgy-created brake pad formulations (BP1, BP2, and BP3). Examine these mate-rials according to their coefficient of friction, noise, vibration, brake force, hardness, and mass loss under varied pressures. Determine the best brake pad composition for various application circumstances by weighing performance parameters including noise, mass loss, and braking force.

## Materials and methods

### Braking pad friction materials

In the present research, powder metallurgy was used to create three semi-metallic brake pad compositions, each consisting of thirteen constituents as explained in Table 1. The following procedures make up the powder metallurgy route: (i) dry mixing; (ii) backing plate preparation; (iii) pre-form compaction; (iv) hot compaction; (v) post-baking; and (vi) finishing. BP1, BP2, and BP3 were the designations assigned to the prototype samples.

**Fig. 2:**
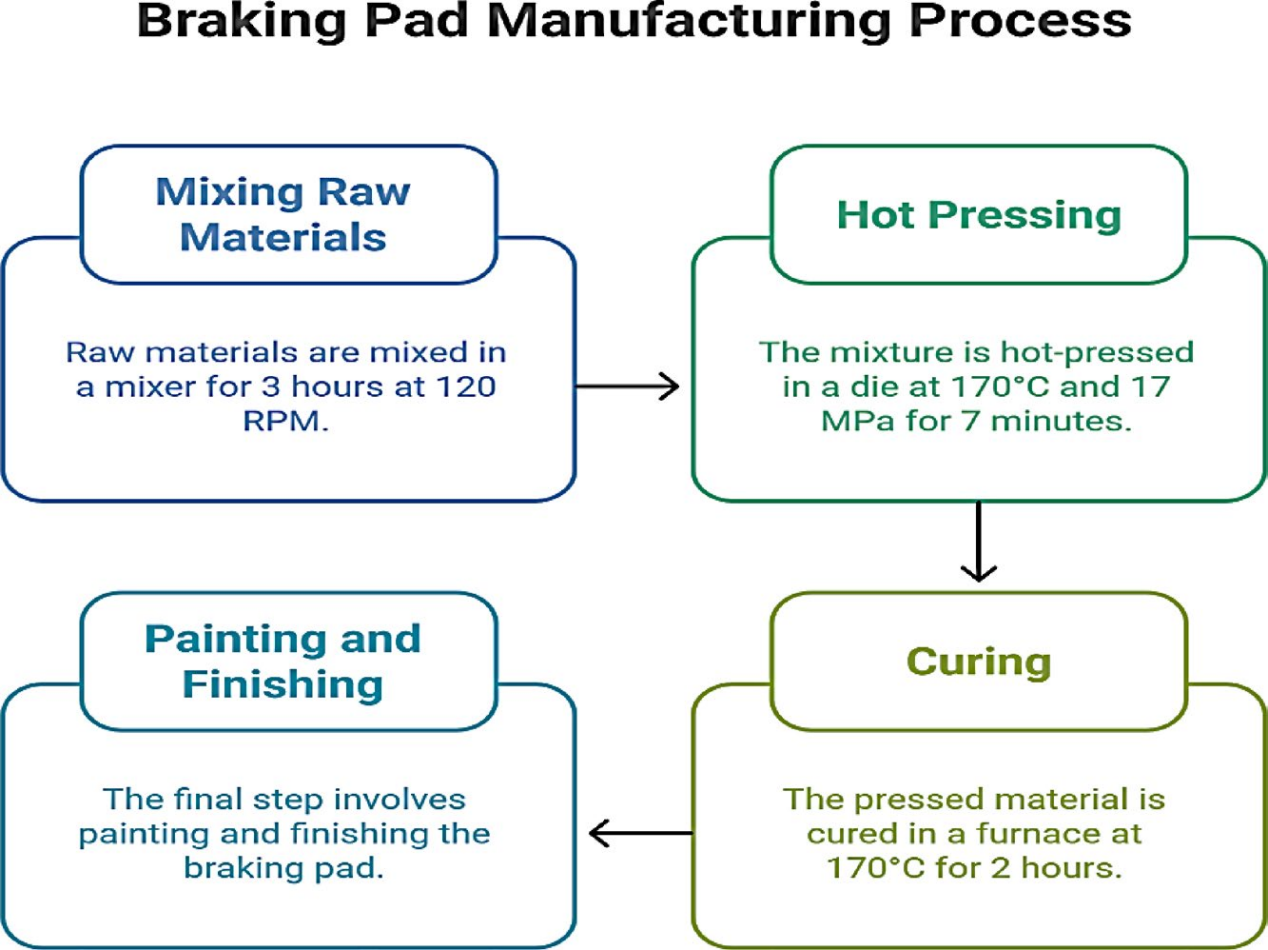
Braking pad manufacturing process.

The mold block, punch, and base made of steel 52 AISI 1055 (equivalent to Ck55, DIN) is used to create the experimental samples for the tested composite frictional material. The proposed samples will be compressed in the mold during the designated curing period (170 ℃, 17 MPa for 7 min). Figure 2 explains braking pad manufacturing process.

The differences between the brake pad formulations BP1, BP2, and BP3 are related to their constituent compositions, which were intentionally varied to explore different performance characteristics:

BP1: This formulation has 13 constituents, including silicon carbide (SiC) and zirconium oxide (ZrO₂), along with a barite content of 26.5%. BP1 was expected to provide high braking force and frictional effectiveness, given the hard and abrasive nature of SiC and ZrO₂, which enhance wear resistance and friction stability. These additives tend to create a stronger, more compact structure, which may increase noise and vibration but also provide durability and high stopping power under stress.

BP2: This variant also has 13 constituents, but it excludes SiC and includes a higher barite content (29.5%) than BP1. Barite serves as a filler that stabilizes friction without high abrasiveness, leading to a more balanced mass loss, moderate noise, and vibration. The absence of SiC was intended to reduce wear and noise while maintaining effective braking force, though not as high as BP1. BP2 aims to provide a balanced performance, suitable for situations where both durability and smooth operation are priorities.

BP3: Like BP2, BP3 has 12 constituents but excludes zirconium oxide and contains the highest barite content at 30.5%. With ZrO₂ removed, the expectation was to achieve a smoother and quieter braking experience with lower friction and braking force compared to BP1. BP3’s high barite content and absence of hard abrasives like ZrO₂ and SiC suggest it would exhibit lower wear and vibration, making it ideal for applications where noise reduction and longevity are valued over maximum stopping power.

**Table 1:** Composition of friction materials braking pads.

No.	Element	Weight%		
		BP1	BP2	BP3
1	Graphite	5.5	5.5	5.5
2	Wire	5	5	5
3	Rock wool	5	5	5
4	Rubber	7	7	7
5	Lime	5	5	5
6	Barite	**26.5**	**29.5**	**30.5**
7	Vermacult	6	6	6
8	Resin	11	11	11
9	Zirconium oxide	4	4	**0**
10	Aramid fiber (3 mm)	7	7	7
11	SiC	3	**0**	3
12	MgO	7	7	7
13	Coke	8	8	8
14	Total	100	100	100

### Mechanical test and microstructure

Specimens of similar diameters to those used for microscopy were utilized to assess hardness. Rockwell C hardness was measured at room temperature using a Zwick/Roell ZHR hardness tester with a diamond indent and a 150 kg load in a 250 × 150 mm^2^ test area. The average of four measurements is used to report each hardness value.

Microstructure samples were characterized using laser scanning confocal microscopy (LSCM, VK - ×200, Keyence Ltd., Osaka, Japan) and a field emission scanning electron microscope (SEM) (FESEM, Carl Zeiss Sigma AG, Oberkochen, Germany).

To determine the volume percentage and lattice parameter of retained austenite, XRD (PAN analytical) was used. Cr K radiation that had not been filtered was used for XRD. Across the angular range of diffracted electrons (2) from 50°−165°, an acceleration voltage of 45 kV and a step size of 0.1° were utilized. The dispersed intensity is measured as a function of outgoing direction when an X-ray beam is pointed at a sample. The angle be-tween the directions of the entering and departing beams is commonly referred to as 2θ.

The crystallographic structure of the friction material extracted from each braking pad sample was examined using the X-ray Diffraction (XRD) test. For this, specialized XRD equipment was used, which made it possible to precisely examine the crystalline phases of the samples. The specimens for the XRD examination were the 1 cm cubes of friction mate-rial, which were carefully set up for optimal regularity.

### Wear test

Test carried out on the suggested specimens using a Pin-on-disc machine to deter-mine each specimen’s mass loss and friction coefficient as condition tests are the disc’s maximum speed is 400 rpm, its pin is positioned at 40 mm in diameter, and the test time 20 min. The brake pad disc is made of gray cast iron, measuring 180 mm in diameter and 25 mm in thickness.

**Fig. 3:**
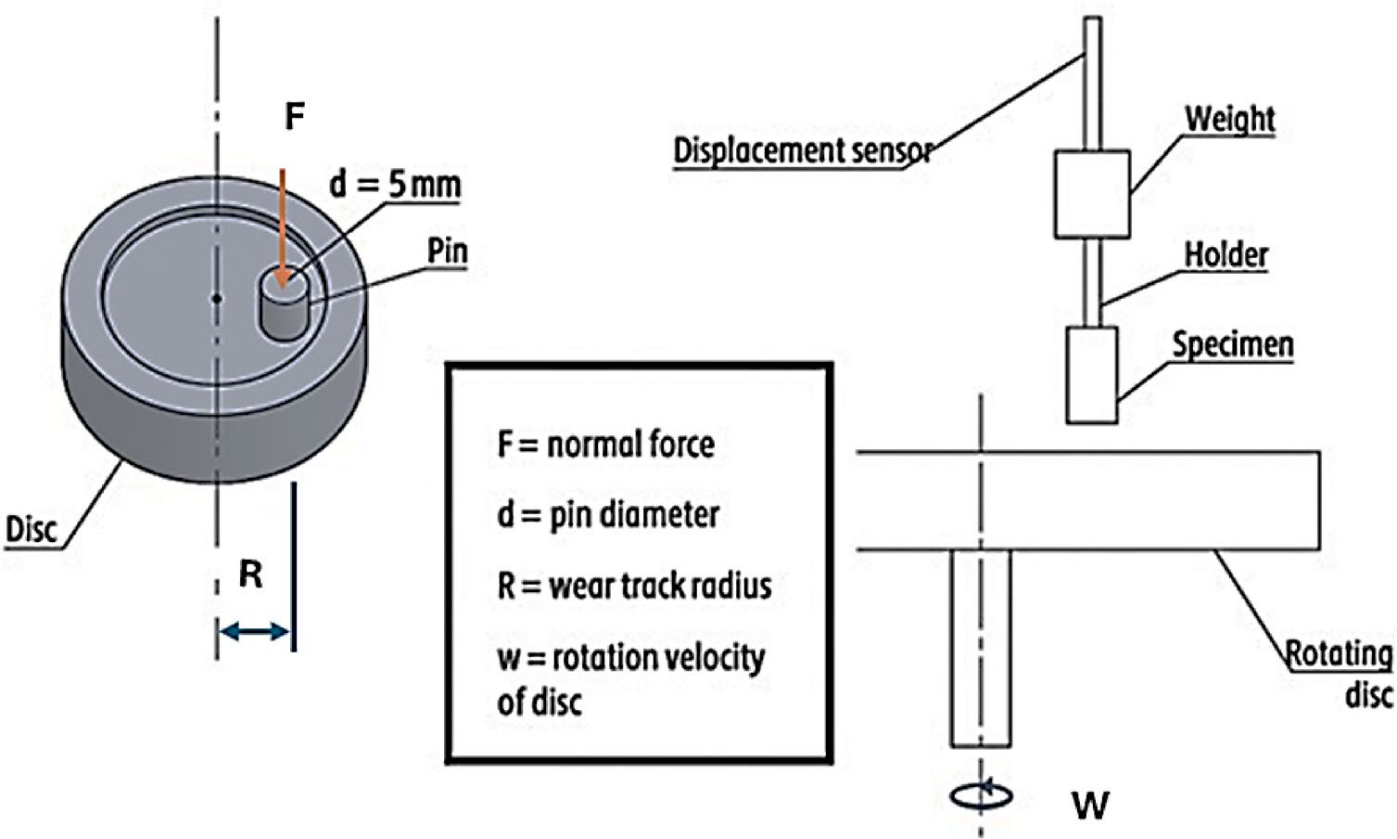
Schematic views of the pin-on-disk apparatus.

The pin-on-disc machine, which measures tribological parameters including mass loss and friction coefficient, has the following requirements:

Getting the necessary measurements ready for a specimen’s pin: height = 21 mm and diameter = 9 mm. Placing the specimen in the machine and rubbing it against the disc at the prescribed load, speed, time, and location, Fig. 3 shows schematic diagram for pin – on - disc test. For 20 min, the reading, which repre-sents an instantaneous coefficient of friction, is recorded every 40 s.

The braking force was measured using a test rig. The test was conducted at two different applied pressures (5 and 8 bar), with a rotating gray cast iron disc (diameter 180 mm, thickness 25 mm) and a brake pad sample pressed against the disc. The rotational speed was maintained at 400 rpm, and the test duration was 20 min for each sample. The braking force was calculated based on the tangential friction force generated during the contact and the applied normal force, with frictional data recorded every 40 s for real-time analysis.

A noise level meter is used to measure the noise level of the proposed frictional composite material specimen.

## Results and discussions

### Microstructure analyses

To assess the distribution of dust in the brake pad composition and investigate deterioration on the brake pad surface, microstructural investigations are carried out. The properties of the brake pad, the suitability of the components, and their uniform distribution within novel formulations are all ascertained using this crucial examination.

**Fig. 4:**
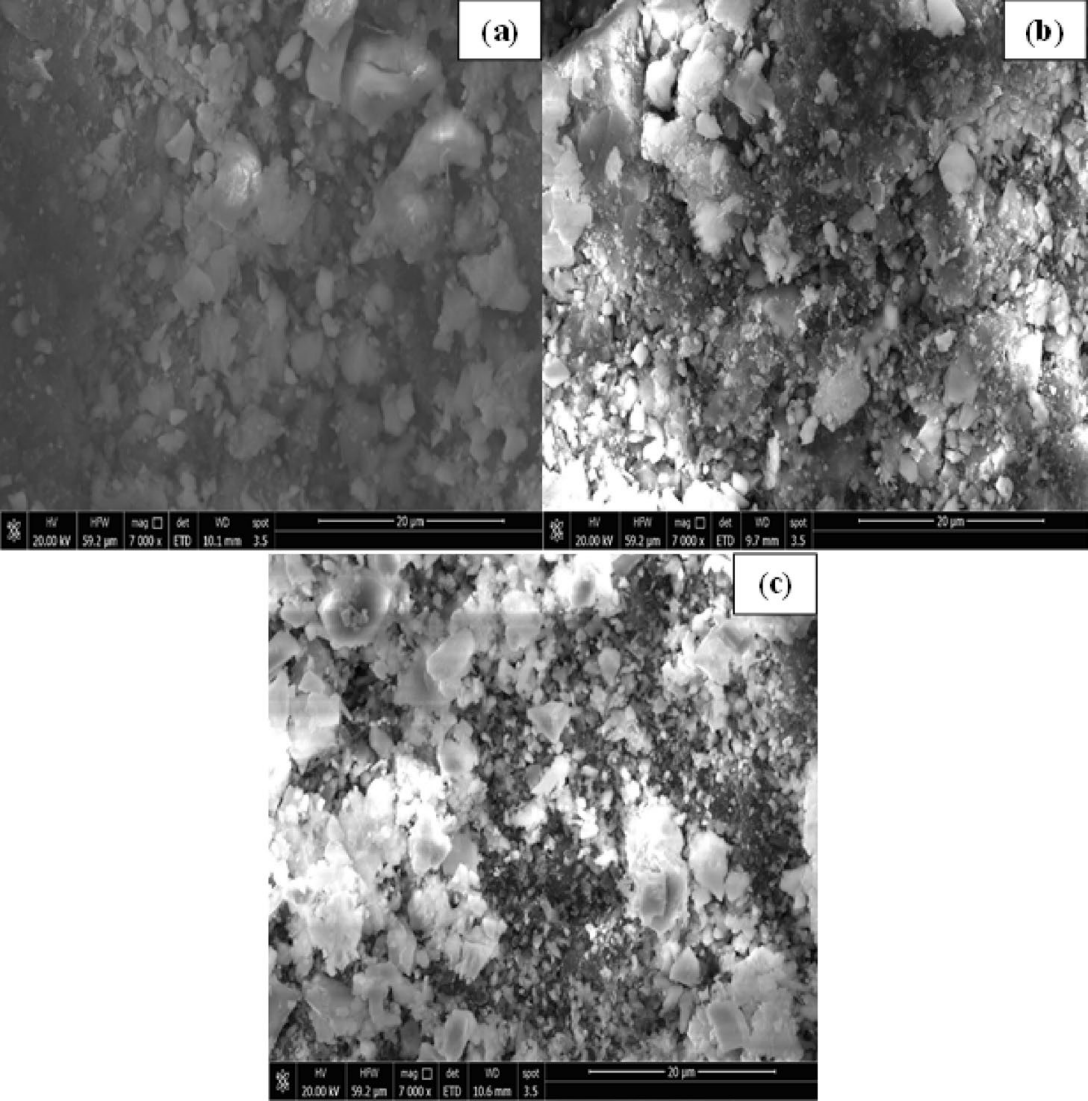
SEM microstructure analyses for samples (**a**) BP1, (**b**) BP2 and (**c**) BP3.

The microstructure of the recently created brake pad is depicted in Fig. 4. According to the earlier images, the specimens’ surfaces are free of cavities and cracks, and the components are distributed uniformly^31^.

With less obvious pores or spaces between particles, the microstructure of BP1 Shows a relatively compact and homogeneous microstructure with reduced visible porosity. The fine distribution of particles and dense packing align with the high hardness and braking force observed in BP1, though also contributing to higher noise and wear due to reduced energy dissipation through pores.

The structure of BP2 is more varied, containing both fine and coarse particles. With some obvious spaces between the particles, BP2 seems to have a somewhat more porous structure than BP1. The particle distribution points to a harmony between porosity and strength, which is consistent with BP2’s mediocre vibration and braking force performance. Because the gaps between the particles may aid in heat dissipation and wear reduction, this structure may help maintain a balanced mass loss.

BP3 Displays a more heterogeneous structure with larger pores and less densely packed particles. The openness of this structure explains the lower mass loss, noise, and vibration characteristics of BP3, as the porous regions help dissipate mechanical stress and heat during braking.

### X-Ray diffraction (XRD)

**Fig. 5:**
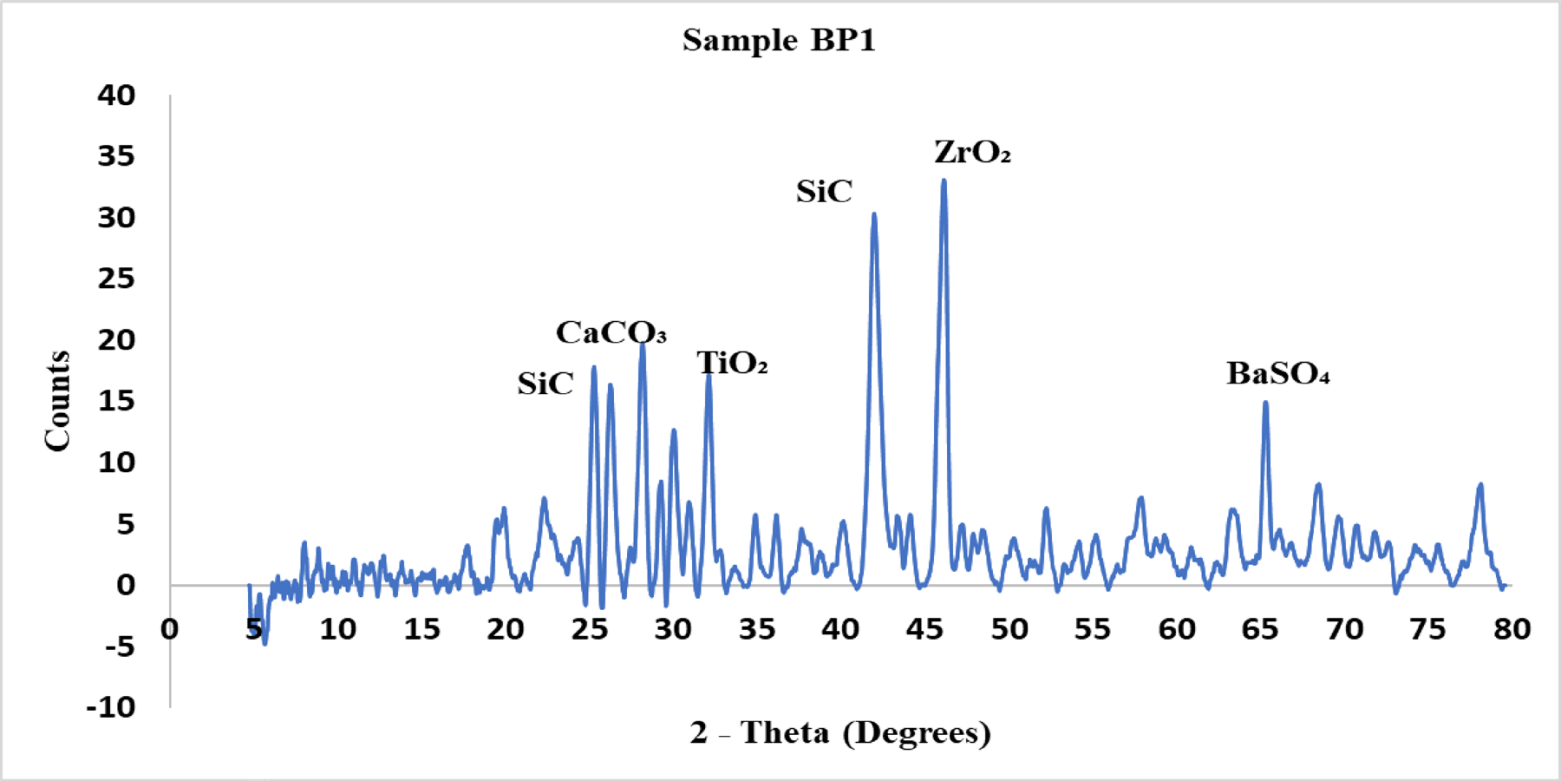
XRD analysis for sample BP1.

**Fig. 6:**
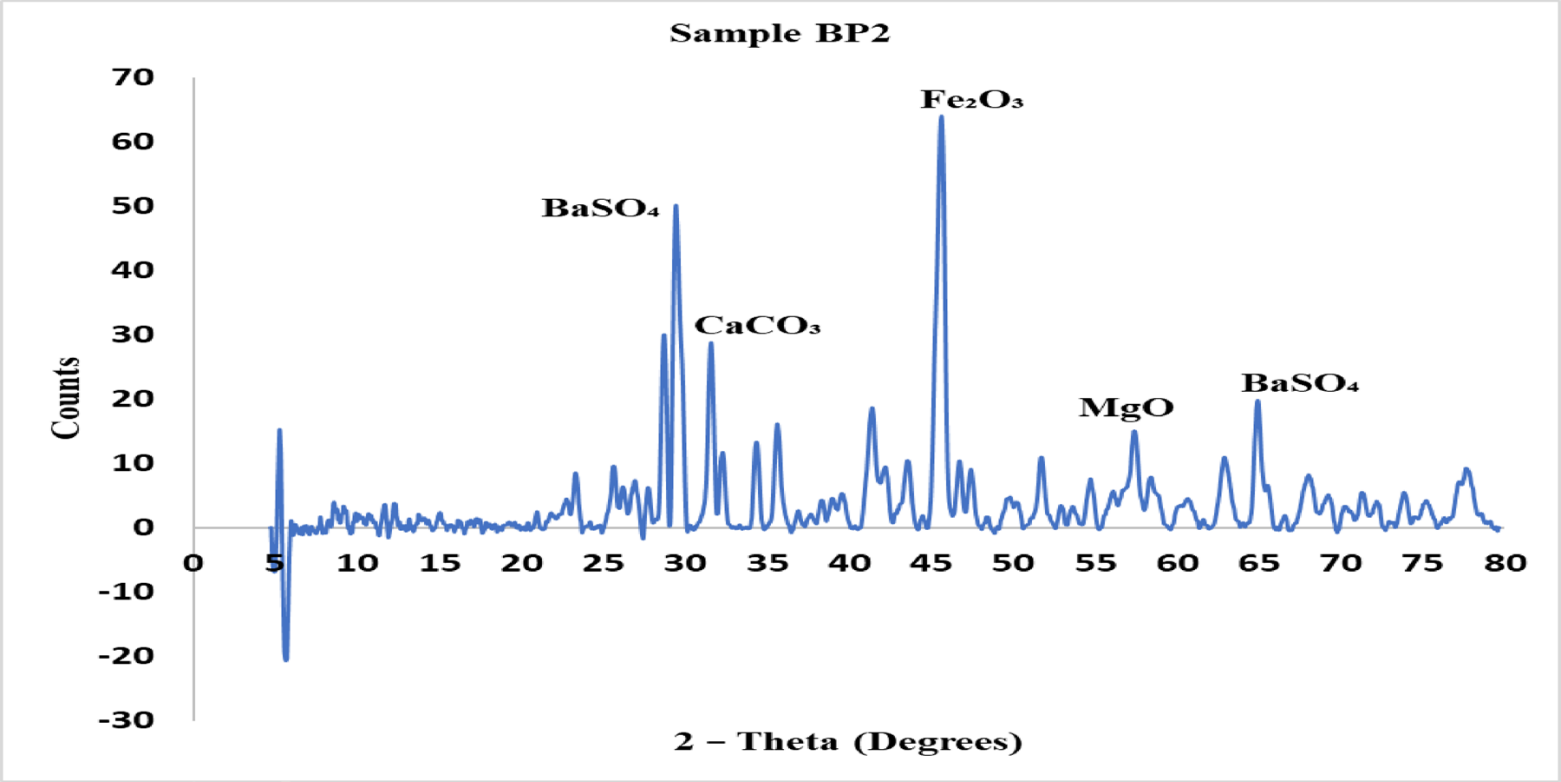
XRD analysis for sample BP2.

**Fig. 7:**
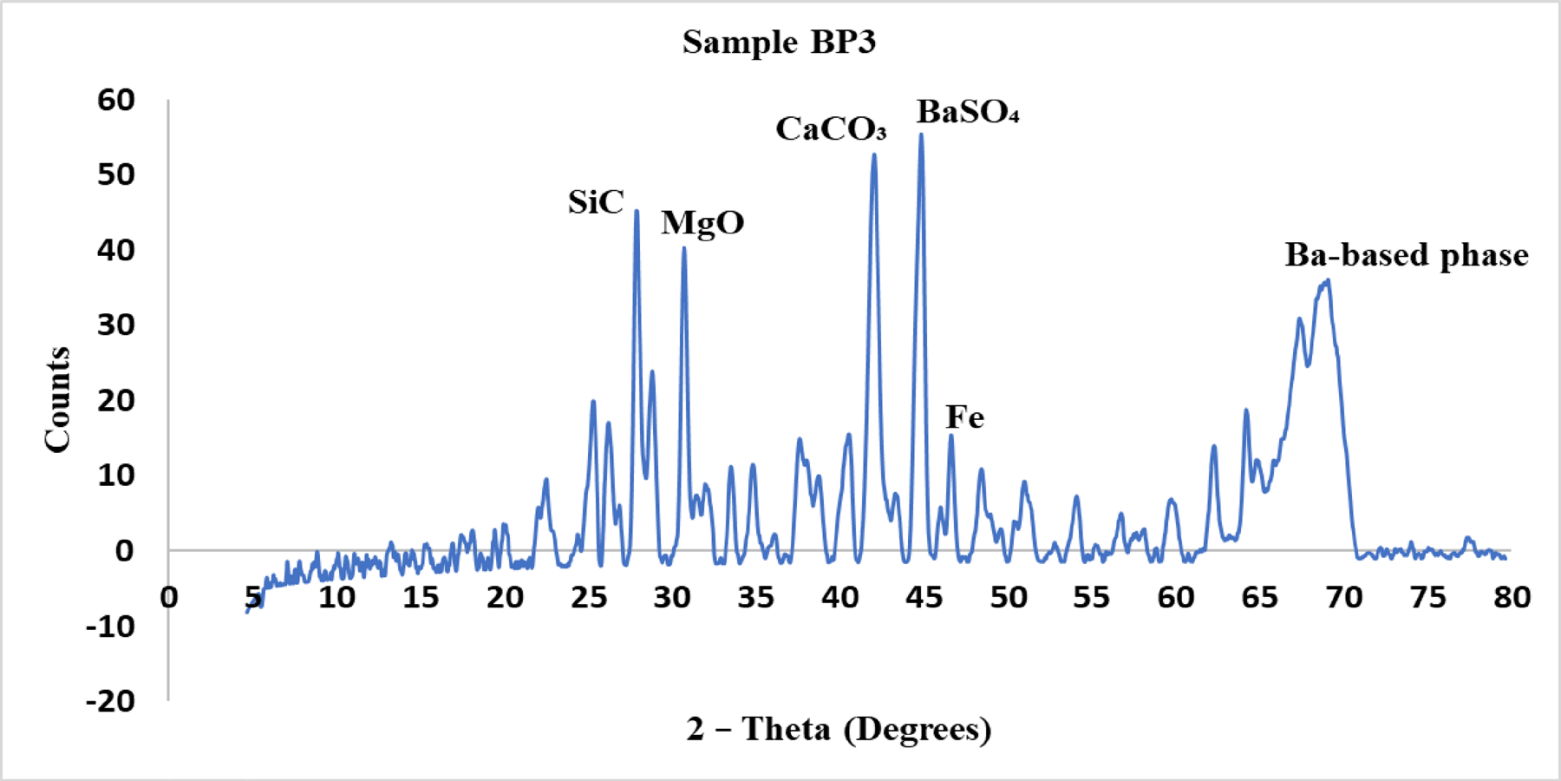
XRD analysis for sample BP3.

The crystalline phases found in each brake pad material are shown by the X-ray diffraction (XRD) patterns for samples BP1, BP2, and BP3 are explained from Figs. 5, 6 and 7. The XRD analysis was carried out to examine the crystalline phases present in the three brake pad formulations BP1, BP2, and BP3. These phases play a critical role in determining the tribological behavior, mechanical strength, wear resistance, and thermal stability of each formulation. The diffraction patterns of the samples revealed distinct peaks corresponding to various compounds, which were identified based on standard ICDD card numbers and matched to known materials relevant to braking applications.

In the case of Sample BP1, the XRD pattern exhibited sharp and intense peaks at approximately 2θ = 26°, 29°, 32°, 38°, 43°, and 45°, indicating the presence of highly crystalline and hard phases. The identified compounds included silicon carbide (SiC), titanium dioxide (TiO₂), calcium carbonate (CaCO₃), zirconium dioxide (ZrO₂), and barium sulfate (BaSO₄). The presence of SiC and ZrO₂, both known for their high hardness and abrasive nature, supports the observed high braking force and hardness of BP1. TiO₂ likely contributes to thermal stability and improved frictional performance, while BaSO₄ and CaCO₃ function as friction stabilizers and fillers. These hard phases collectively enhance wear resistance and braking effectiveness but may also lead to increased noise and vibration.

For Sample BP2, the XRD pattern showed peaks of moderate intensity at similar 2θ values but with a noticeable absence of the very sharp peaks observed in BP1. The key identified phases were BaSO₄, CaCO₃, MgO (magnesium oxide), and Fe₂O₃ (hematite). These compounds suggest a more balanced composition with a mix of crystalline and semi-amorphous materials. The absence of hard reinforcements like SiC and ZrO₂ indicates that BP2 is designed for moderate hardness and reduced abrasiveness. MgO serves as a mild abrasive that contributes to wear regulation, while Fe₂O₃ enhances oxidation resistance. This combination supports the observation that BP2 has intermediate properties between BP1 and BP3 offering adequate braking performance with reduced noise and wear.

Sample BP3 presented a broader distribution of peaks with generally lower intensities, indicating a higher proportion of amorphous or loosely packed crystalline phases. The main compounds identified in the XRD pattern include SiC, MgO, CaCO₃, BaSO₄, iron (Fe), and a barium-based compound evident from a broad peak near 70°. Unlike BP1, BP3 lacks TiO₂ and ZrO₂, which suggests a softer and more compliant microstructure. The presence of BaSO₄ and CaCO₃ ensures frictional stability, while the reduced intensity of SiC implies lower abrasiveness. The iron phase and MgO contribute to structural strength and mild friction enhancement. The Ba-based compound observed at high angles likely improves thermal stability while maintaining low noise and vibration. These structural characteristics align with BP3’s performance data, which indicate lower wear, noise, and vibration making it more suitable for long-life and comfort-focused applications^2,13^.

### EDAX analysis

**Fig. 8:**
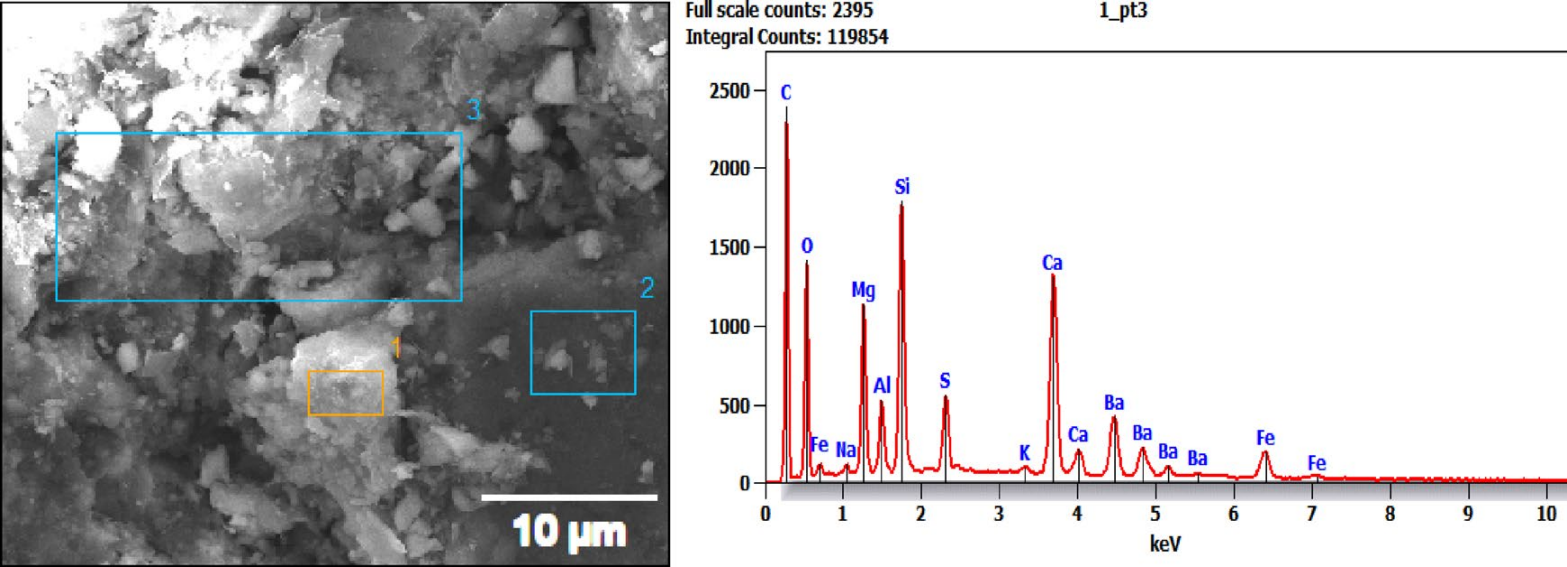
EDAX Position at area 3 for sample BP1.

**Fig. 9:**
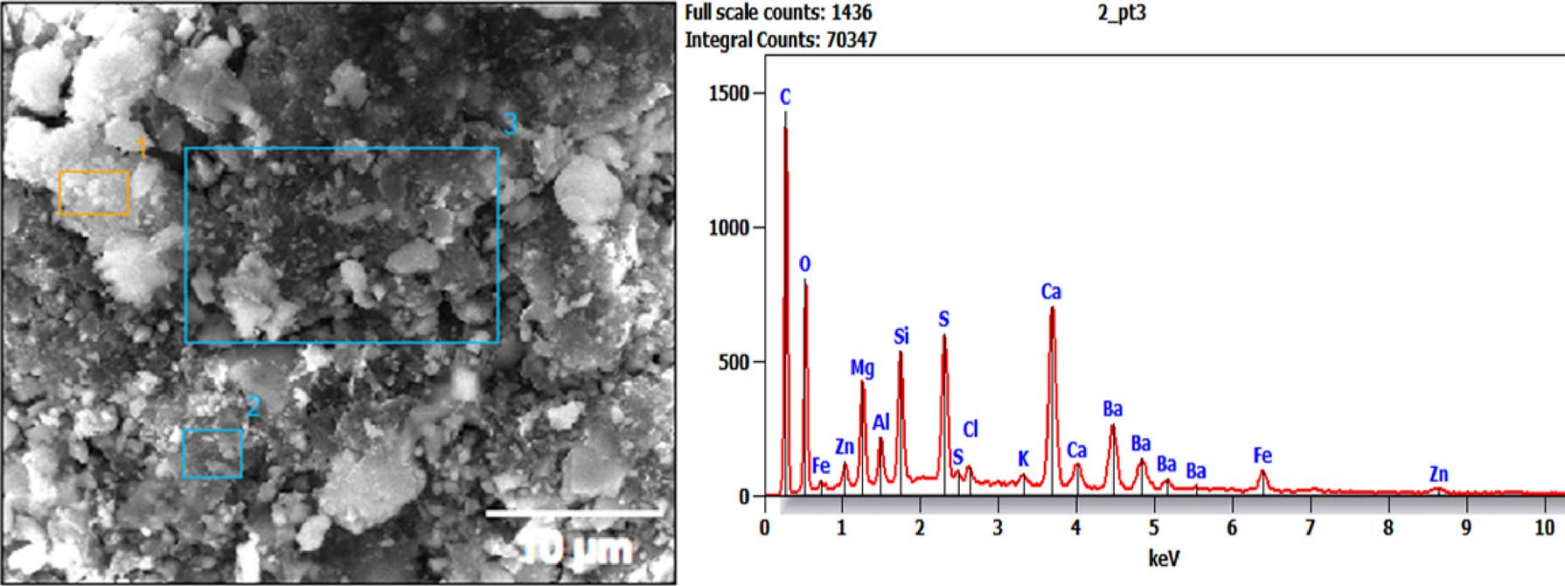
EDAX Position at area 3 for sample BP2.

**Fig. 10:**
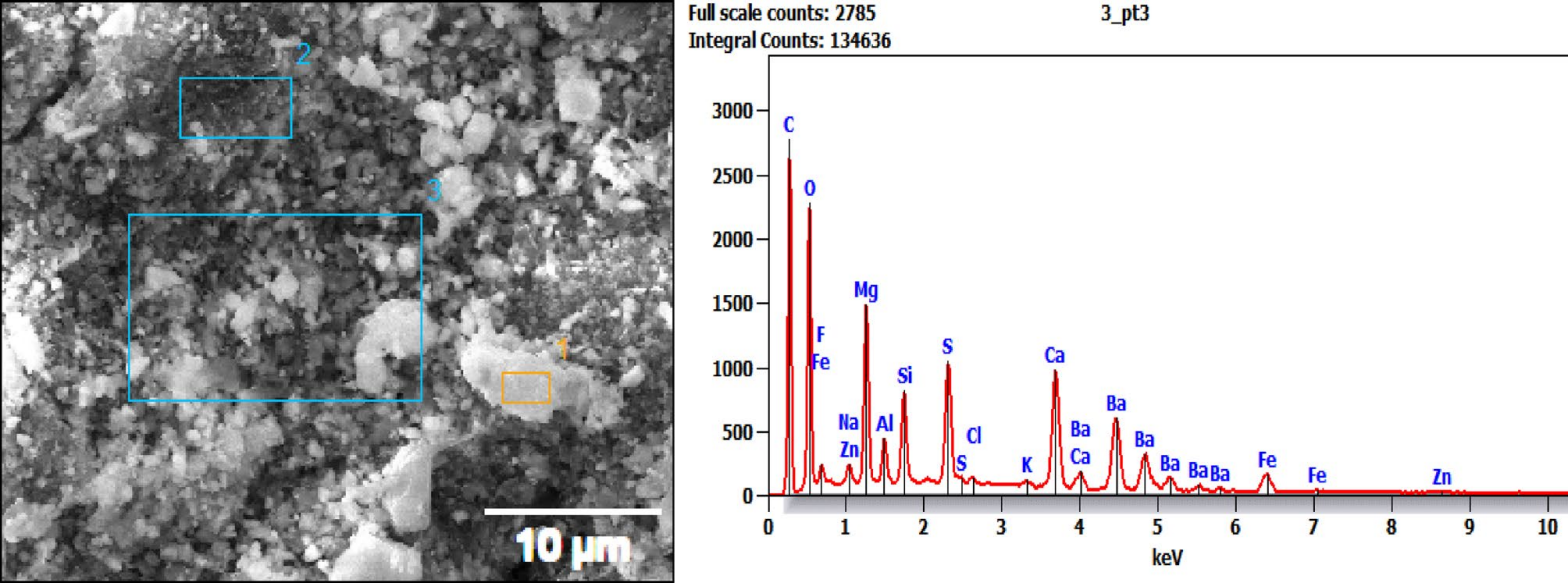
EDAX Position at area 3 for sample BP3.

The elemental composition data associated with the EDX analysis of different regions on samples BP1, BP2, and BP3 is displayed in Table 2, which also identifies the particular elemental differences in each brake pad sample^41^. Here is a comparison and analysis of these findings, emphasizing important components and how they affect brake pad performance Fig. 8 to 10 demonstrates EDAX analysis.

BP1: Perfect for high-performance brakes with high friction, BP1’s high silicon and titanium concentration in specific regions adds hardness and wear resistance. However, because these materials are abrasive, increased wear and noise might be anticipated.

BP2: A well-balanced composition with constant iron and sulfur levels, lower silicon, and moderate quantities of calcium and barium. This makes BP2 adaptable by promoting a balance between wear resistance and improved braking performance.

BP3: Provides smoother, low-vibration braking with durability by emphasizing a high carbon and barium content and a low silicon and calcium concentration. The BP1 sample contains high levels of silicon (Si), calcium (Ca), and barium (Ba), with notable concentrations of iron (Fe) and titanium (Ti) in particular regions.BP1 is appropriate for applications needing high braking force and durability under stress because of its high levels of silicon and titanium, which also contribute to its hardness and wear resistance. Although the composition of BP1 promotes excellent frictional performance, it’s harder, more abrasive components may lead to greater wear and noise.

Barium (Ba), calcium (Ca), sulfur (S), and iron (Fe) are all moderately present in the BP2 sample, whereas silicon (Si) is lower than in the BP1 sample. Moderate wear resistance and frictional stability are supported by BP2’s well-balanced mixture of organic and inorganic components. The BP3 sample exhibits moderate levels of calcium (Ca) and barium (Ba) in every region, along with high levels of carbon (C) and oxygen (O).

The data in the figures show an example of area number 3 only. Elements including carbon (C), oxygen (O), magnesium (Mg), aluminum (Al), silicon (Si), sulfur (S), calcium (Ca), and iron (Fe) exhibit notable peaks in the EDAX spectrum for BP1. There are also faint peaks for barium (Ba), potassium (K), and sodium (Na).With peaks for carbon, oxygen, magnesium, aluminum, silicon, sulfur, calcium, iron, and other elements like chlorine (Cl) and zinc (Zn), the second formulation, BP2, displays a comparable overall composition. Like the second formulation, the third formulation, BP3, displays peaks for carbon, oxygen, magnesium, aluminum, silicon, sulfur, calcium, and iron, along with a few tiny peaks for zinc. Carbon Content: The high carbon content of all three formulations suggests the inclu-sion of organic components that are probably utilized as frictional modifiers or binders.Barium appears consistently in all three formulations, suggesting that it plays a cru-cial role in these pads’ frictional characteristics, perhaps as a filler to improve stability in a range of frictional situations.

The addition of zinc and chlorine in the second and third formulas indicates a little modification aimed at enhancing wear resistance and thermal stability. The third formulation appears to include a relatively higher iron content, potentially influencing its wear characteristics^42^.

**Table 2:** Elemental composition for EDAX results for tested samples.

Element (Wt %)																
Sample	C	N	O	Na	Mg	Al	Si	S	K	Ca	Ti	Fe	Ba	Ci	Zn	F
**BP1** (Area 1)	26	…….	25.4	0.2	3.9	1.6	10	1.9	0.2	14.8	13.5	2.5		…….	…….	…….
**BP1** (Area 2)	13.1	4.1	15.5	…….	1.1	2.8	56.7	0.6	…….	2.5	…….	1	2.6	…….	…….	…….
**BP1** (Area 3)	21.9	…….	20.2	0.4	6	2.4	9.6	3	0.3	13.2	…….	5.9	17.1	…….	…….	…….
**BP2** (Area 1)	33.9	…….	19	…….	6.3	1.1	6.1	5.1	0.4	5.9	…….	3.3	16.2	0.4	2.4	
**BP2** (Area 2)	13.8	…….	22.8	…….	5.2	1.3	3.7	4	0.2	23.4	…….	7.6	17		1	
**BP2** (Area 3)	23.2	…….	21	…….	4.1	1.6	4.7	6.2	0.5	13	…….	4	17.9	0.7	3.1	
**BP3** (Area 1)	31.2	…….	30.9	0.7	5.5	1.9	3.2	3.3	0.2	5.4	…….	4.4	13.4	…….	…….	…….
**BP3** (Area 2)	27.8	…….	20.6	1.1	5.8	1.6	3.1	5.1	0.3	7.6	…….	4.6	20.1	0.3	0.7	1.2
**BP3** (Area 3)	21.4	…….	23.8	0.9	7.2	1.5	3.5	5.4	0.3	8.1	…….	4.1	21.8	0.4	1.1	0.5

### Hardness results

The brake pad samples BP1, BP2, and BP3’s Rockwell Hardness (HRC) values provide information about their material hardness, which affects durability, braking performance, and wear resistance, Table 3 summarize values of hardness.

For the three samples, BP1 has the highest hardness value, indicating that it is an extremely hard and abrasive substance. According to earlier analyses, BP1 performs well as a high-friction brake pad because of its high hardness. Increased wear resistance and braking force are typically correlated with higher hardness. However, because of the abrasive character of the material, this also suggests that BP1 would be more likely to cause increased rotor wear and noise.BP2 appears to be less abrasive than BP1 and BP3 based on its lowest hardness measurement of 38 HRC. BP3’s high hardness value 44 HRC is little lower than BP1’s.

**Table 3:** Hardness measurements.

Sample Number	Rockwell Hardness (HRC)
BP1	46
BP2	38
BP3	44

### Braking force

**Fig. 11:**
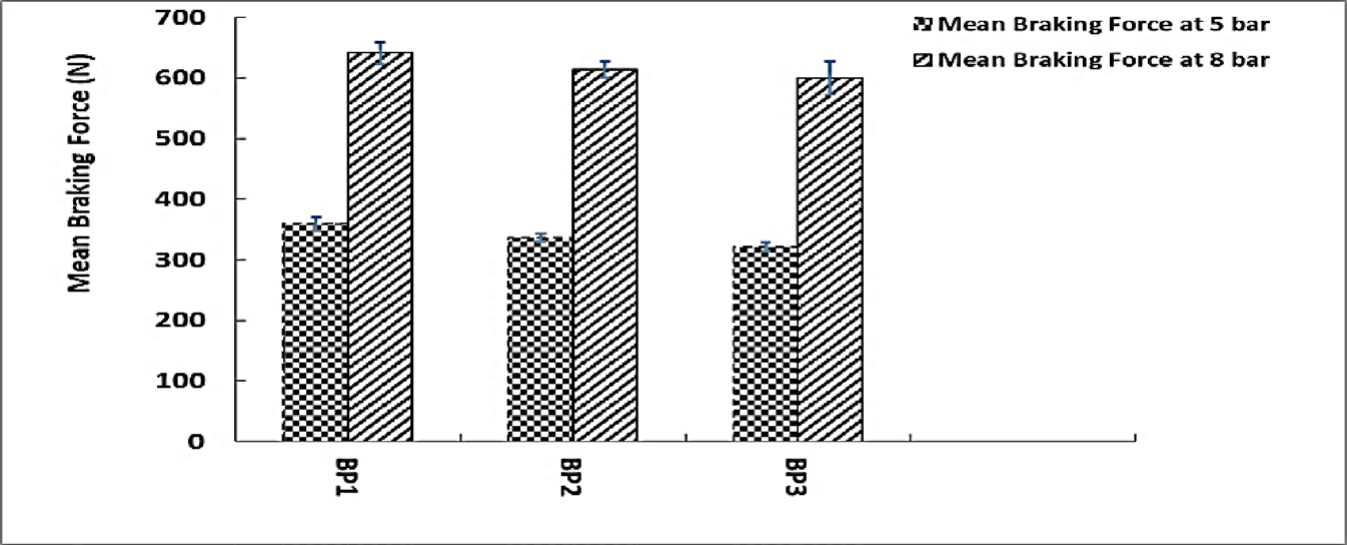
Mean braking force at 5 and 8 bar.

This analysis and discussion of the mean braking force at 5 and 8 bar pressures across three brake pad samples BP1, BP2, and BP3 is based on the data shown in the Fig. 11.

At 5 Bar, the samples’ mean braking forces differ; BP1 had the greatest value 359.4 N, followed by BP2 336.68 N and BP3 320.95 N. BP1 once more exhibits the largest braking force 640.99 N at 8 Bar, followed by BP2 614.1 N and BP3 599.94 N.

As the pressure increases from 5 bar to 8 bar, the braking force for each sample exhibits a significant rise. All samples demonstrate this trend, confirming that increased braking force results from elevated applied pressure. This pattern indicates that brake pads exhibit greater efficiency in generating stopping power at elevated pressures, as increased pressure facilitates larger frictional forces^43^.

The fact that BP1 continuously produces the most braking force at both pressures raises the possibility that it is the most force-producing brake pad of the three. The fact that BP3 continuously exhibits the lowest braking force suggests that, in comparable circumstances, it may offer the least stopping power.

The research results reflect the general idea that higher pressure improves braking performance by showing that all brake pads increase their braking force as pressure increases. Since it constantly achieves the highest braking force, BP1 is clearly the best performer. Since of this, BP1 might be a better option for applications that need more stopping power, particularly when higher pressures are required. Higher pressure also causes BP2 and BP3 to exhibit greater braking force; however, BP3 continuously produces the least force, indicating that it could not be as effective as BP1 and BP2. Since BP3 may result in less wear and smoother performance, it may be taken into consideration for situations where a lower braking force is desired or acceptable. With BP1 being the recommended option for highest braking force, these insights are helpful when choosing brake pads depending on performance requirements^44^.

### Noise and vibration analysis

**Fig. 12:**
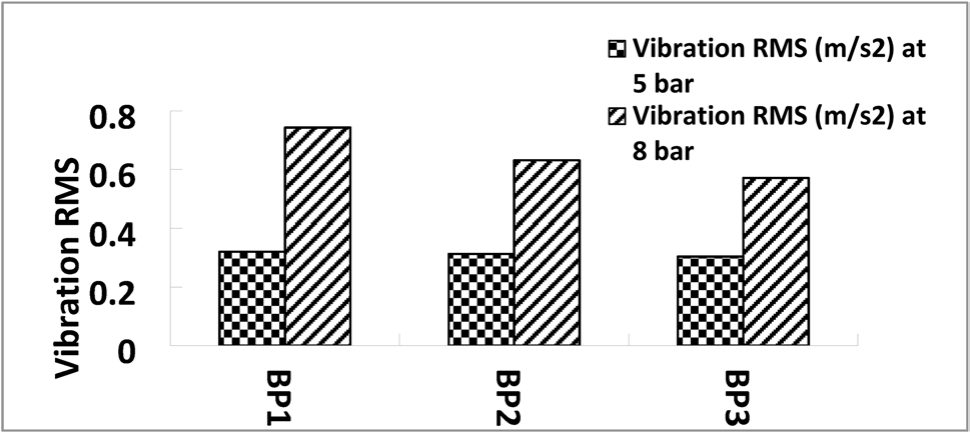
Vibration RMS (m/s^2^) at 5 and 8 bar.

Harder or more frequently occurring vibrations are represented by higher Root Mean Square (RMS) values, which raise micro-movements and frictional energy dissipation at the brake pad surface, Fig. 12 demonstrates RMS results.

Since of the repetitive application of tiny, varying stresses, these frequent vibrations accelerate the wear of the pad material. In essence, wear is accelerated by increased mechanical stress on the pad’s contact surface^45^.

Increased heat during braking is frequently the result of larger braking forces delivered unevenly, which is shown by elevated RMS values. Heat accumulation may cause the brake pad material to deteriorate thermally, hastening wear. Additionally, the heat may cause the pad material to harden or develop “glazing,” which lowers braking efficacy and necessitates a higher RMS in order to provide efficient braking. Wear is further accelerated by this feedback loop.

Lower wear is generally correlated with diminished RMS values, signifying more uniform and consistent braking forces. Conversely, elevated RMS values are often associated with more severe braking situations and accelerated material deterioration. Consequently, prolonging pad longevity and improving braking comfort and efficiency can be accomplished by optimizing brake systems to sustain reduced RMS values^43^.

The RMS vibration value of BP1 is the highest at 0.321 m/s², followed by BP2 at 0.313 m/s² and BP3 at 0.304 m/s². These minor variations suggest that, at lower pressures, the samples’ vibration levels are very similar, with BP1 exhibiting somewhat greater vibration^10,46^.

The RMS vibration values for each sample likewise rise noticeably as the pressure reaches 8 bar. The highest RMS value is still 0.743 m/s² for BP1, which is followed by 0.631 m/s² for BP2 and 0.571 m/s² for BP3.Since higher pressure usually results in larger frictional forces, which in turn cause more vibration, the rise in vibration with pressure is to be expected.

At both pressures, BP1 exhibits the largest RMS vibration, indicating that it generates vibrations with greater intensity. Higher wear potential and less comfort as a result of harder braking could result from this. At both pressures, BP3 has the lowest RMS vibration, indicating smoother braking, which could improve customer service and lessen brake system wear.

Since more applied force results in a more intense interaction between the brake pad and rotor, the data shows that all brake pad samples show increased vibration RMS with higher pressure. According to earlier tests, BP1, which has the greatest vibration RMS, may provide good braking performance, but at the expense of increased vibration, which may result in more wear and perhaps affect driver comfort. With the lowest vibration RMS values, BP3 is anticipated to offer the smoothest braking experience, making it appropriate for uses where durability and comfort are top concerns. In the middle, BP2 provides a harmony between comfort and performance.

**Fig. 13:**
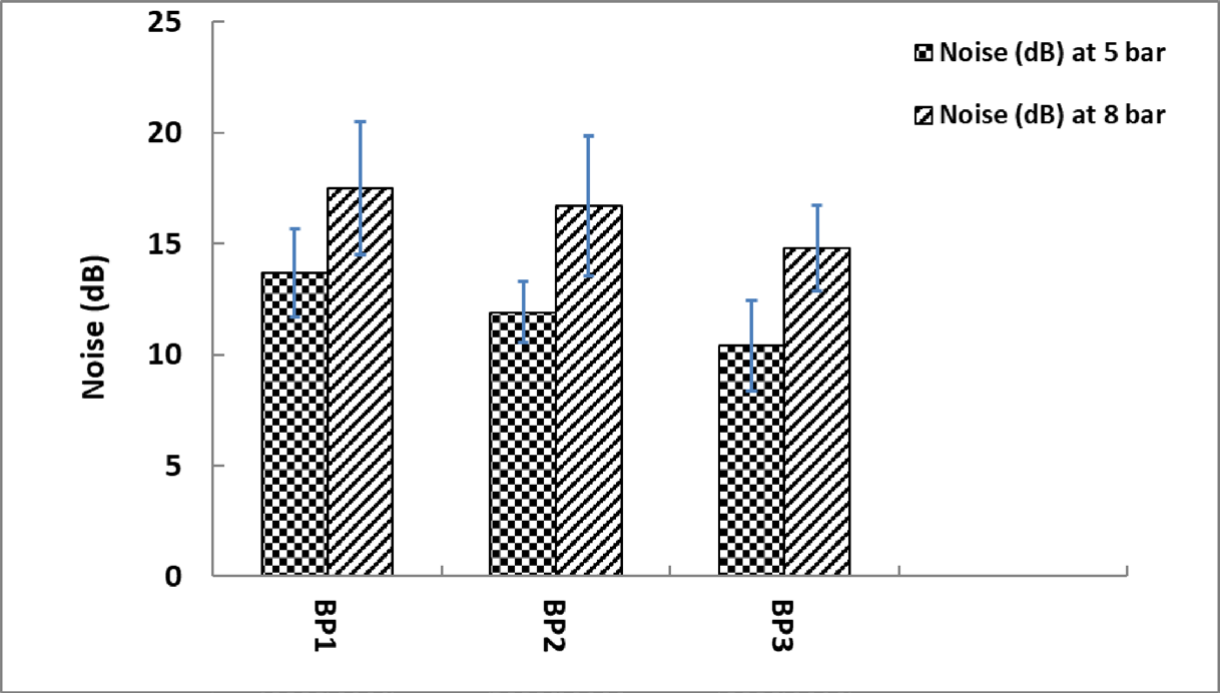
Noise results for tested samples.

Figure 13 shows noise level values. The highest noise level at 5 Bar is 13.7 dB for BP1, 11.9 dB for BP2, and 10.4 dB for BP3.This pattern suggests that BP1 is a higher-friction pad, frequently linked to louder braking, because it produces more noise even at lower pressures^45^. All samples experience higher noise levels at 8 bar, which is to be expected as higher pressure tends to amplify noise because of increased friction and vibration. At 17.5 dB, BP1 once more records the greatest noise level, followed by BP2 16.7 dB and BP3 14.8 dB.

At both pressures, BP1 has the highest noise levels, indicating that it might not be a suitable option for applications where noise reduction is essential. With the lowest noise levels at both pressures, BP3 might be a better fit for applications that prioritize comfort and quiet operation.

As the pressure increases, the data clearly demonstrates an increase in noise, which is common in braking systems because of increased friction forces. In line with its strong braking force and vibration characteristics noted in earlier investigations, BP1 continuously has the highest noise levels. This implies that although BP1 might provide excellent braking, it does so at the price of increased noise and possibly decreased comfort. Since BP3 has the lowest noise levels and seems to be the quietest of the samples, it would be a better choice for applications that prioritize user comfort and minimal noise emissions, including quiet environments or passenger cars. In the middle, BP2 offers a reasonable trade-off between noise and performance.

### Coefficient of friction analysis

**Fig. 14:**
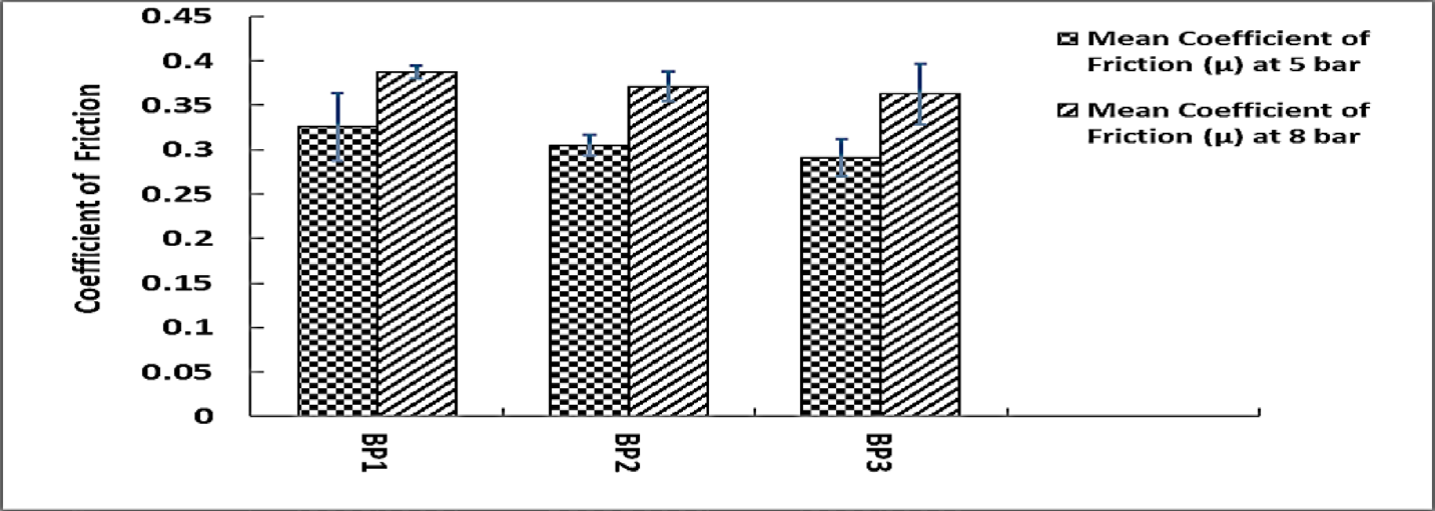
Mean coefficient of friction for tested samples.

The samples have somewhat different mean coefficients of friction at 5 bars; BP1 has the greatest value 0.3257, followed by BP2 0.305 and BP3 0.291. Similar trends are seen at 8 Bar, with BP1 displaying the highest coefficient once more 0.3873, followed by BP2 0.371 and BP3 0. 3626.As the pressure is increased from 5 bar to 8 bar, the coefficient of friction rises for every sample^10^. The fact that this rise is constant across BP1, BP2, and BP3 suggests that the brake pads have superior grip when pressure is increased. According to this pattern, higher pressure improves the contact between the braking surface and the brake pad material, which improves braking efficiency. Figure 14 shows COF values.

In comparison to BP2 and BP3, BP1 consistently exhibits the highest coefficient of friction, indicating superior material properties for frictional performance. Consistently exhibiting the lowest coefficient of friction, BP3 may demonstrate inferior performance regarding friction under similar conditions^47^.

According to the data, all samples exhibit better frictional performance at greater pressures, indicating that the brake pads react favorably to increased applied pressure. In braking systems, where more applied pressure usually results in stronger stopping power, this behavior is consistent with predictions. Though BP3 may wear more readily under high-stress situations because of its comparatively lower friction coefficient, the differences between the samples suggest that BP1 may provide the best overall friction performance.

**Fig. 15:**
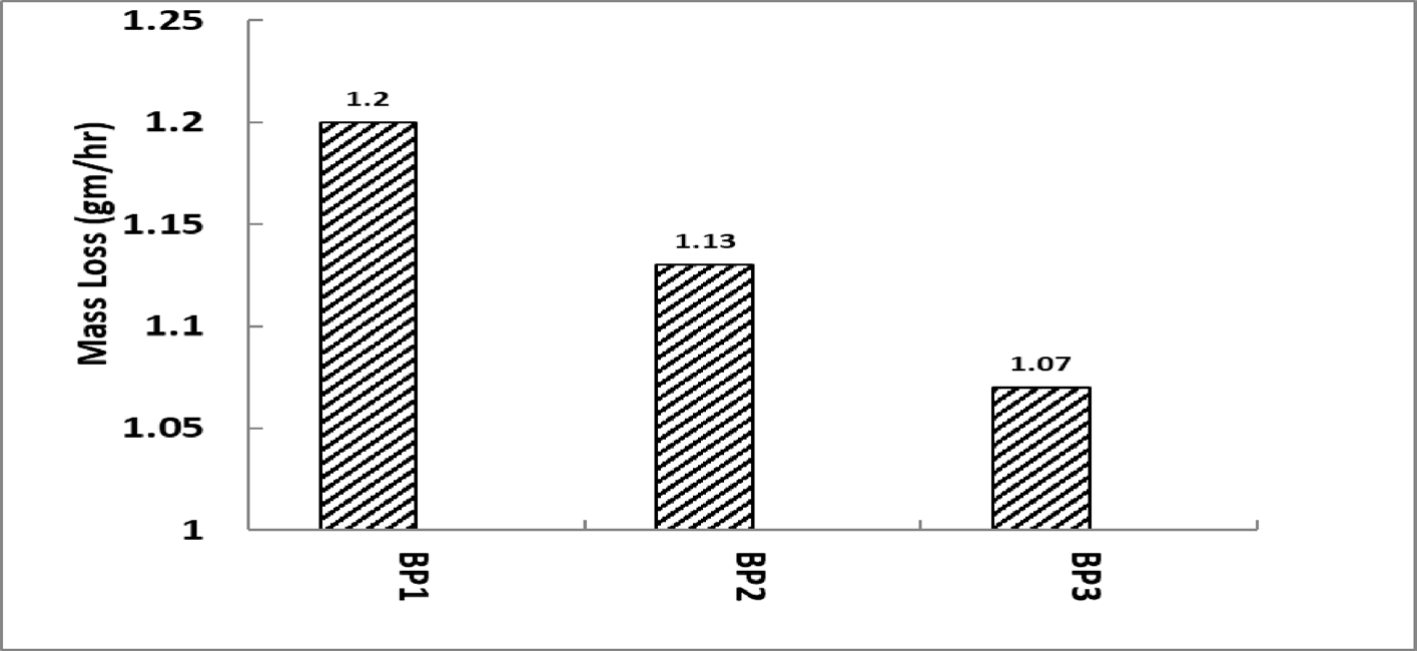
Mass loss for tested samples.

A higher mass loss indicates that the material used to make brake pads deteriorates more quickly under the same circumstances, thus requiring more frequent replacements and higher maintenance expenses, Fig. 15 shows mass loss for tested samples^45^. According to earlier research, BP1 may offer greater brakes at the cost of faster wear because of its higher mass loss, higher braking force, and vibration RMS. This is common for brake pads made with stopping power rather than longevity in mind. The pad with the lowest mass loss, BP3, is perhaps the most resilient of the three. This is consistent with BP3’s lower vibration RMS, which points to a smoother functioning as well as less material stress. Table 4 summarizes the results.

**Table 4:** Table summary of the results.

Mechanical Characteristics		BP1	BP2	BP3
Hardness (HRC)		46	38	44
Mean Braking Force (N)	5 Bar	359.4	336.68	320.95
	8 Bar	640.99	614.1	599.94
Root Mean Square (RMS)	5 Bar	0.321	0.313	0.304
	8 Bar	0.743	0.631	0.571
Noise (dB)	5 Bar	13.7	11.9	10.4
	8 Bar	17.5	16.7	14.8
Coefficient of Friction (COF)	5 Bar	0.3257	0.305	0.291
	8 Bar	0.3873	0.371	0.3626
Mass loss (gm/h.)		1.2	1.13	1.07

## Conclusion

Significant variations in performance across the three brake pad compositions were revealed by our analysis:

- The BP1, BP2, and BP3 brake pad formulations’ XRD results revealed variations in their crystalline and amorphous phase distributions, which had an immediate effect on their mechanical characteristics: BP1 showed prominent peaks at particular 2-theta values (30°, 45°, and 50°), suggesting the presence of extremely stable crystalline phases such as titanium oxide (TiO₂) and silicon carbide (SiC). Its better wear resistance and frictional performance were consistent with the high-intensity peaks indicating increased hardness and stability.
- BP2 exhibited a mixture of crystalline and amorphous phases with moderate peak intensities in comparison to BP1. Compounds like barium sulfate (BaSO₄) and calcium carbonate (CaCO₃) were used to develop a material that is balanced in terms of frictional stability and wear resistance without being overly abrasive.
- Wider peak distributions, with notable peaks at 30° and 70°, characterized BP3, suggesting a combination of softer amorphous and stable crystalline phases. This mixture is appropriate for less-abrasive and quieter applications because it has reduced hardness and improves longevity while retaining enough frictional stability.
- According to the EDAX, the elemental bases of all three formulations were comparable and included carbon, oxygen, silicon, calcium, sulfur, aluminum, and barium—all of which are common in braking pad compositions to offer stability, longevity, and friction management. Nonetheless, the minor variations in the amounts of iron, zinc, and chlorine in the formulations pointed to the need for focused modifications to maximize qualities, such as wear resistance, thermal stability, or frictional behavior, based on the use of each brake pad.
- At both 5 and 8 bar, BP1 continuously generated the greatest braking force, with values of 359.4 N and 640.99 N, respectively. At 5 bar and 8 bar, BP2 demonstrated moderate braking forces of 336.68 N and 614.1 N, respectively. The application of BP3 in high-force situations may be limited because BP3 had the lowest braking force, measuring 320.95 N at 5 bar and 599.94 N at 8 bar.
- Coefficient of friction and braking force: At 8 bar, BP1 continuously demonstrated the highest coefficient of friction and braking force, higher by 10% over BP2 and 15% over BP3. Because of this, BP1 is appropriate for uses where a high stopping power is needed.
- Mass loss: As a sign of increased durability, BP1 had the highest mass loss, followed by BP2, and BP3 had the lowest mass loss. Compared to BP1, BP3’s mass loss was about 20% lower, indicating that BP3 would be more affordable in terms of longevity and maintenance.
- Noise and vibration: At the maximum pressure, BP1 had the highest noise and vibration levels, around 15% higher than those of BP2 and 25% higher than those of BP3. The low vibration and noise levels of BP3 suggest that it is appropriate for applications that prioritize silent operation and user comfort.
- Hardness: BP1 had the highest hardness at 46 HRC, followed by BP3 at 44 HRC and BP2 at 38 HRC, according to the hardness measurements. Although BP1’s higher hardness was related to its improved frictional stability, it also showed increased wear and noise.

## Data Availability

Data Availability: The datasets used and/or analyzed during the current study are available from the corresponding author on reasonable request.
